# A qualitative exploration of parental perspectives and behaviors on self-medication for children under five in Abbottabad, Pakistan

**DOI:** 10.3389/fped.2025.1445219

**Published:** 2025-04-16

**Authors:** Ijaz ul Haq, Syed Hanzila Azhar, Shahbaz Ahmad Zakki, Xiaojing Hu

**Affiliations:** ^1^ Nursing Department, Children's Hospital of Fudan University, Shanghai, China; ^2^ Department of Public Health & Nutrition, The University of Haripur, Haripur, Pakistan

**Keywords:** parental self-medication, under five-year child, thematic analysis, qualitative study, health seeking behavior

## Abstract

**Background:**

Children are more susceptible to irrational medication and its short- and long-term health consequences. This study investigated parents' perceptions and behavior regarding self-medication of their children under five years of age in city Abbottabad, Pakistan.

**Methods:**

Non-probability convenient random selection was used to select participants for this qualitative study. In-depth interviews were conducted with 40 parents who self-medicate their children, utilizing audio recordings and note-taking as the methods for data collection. N-Vivo 11.0 was used for thematic analysis and data has presented according to COREQ checklist.

**Result:**

The majority (45%) of parents were aged 25–35 years, while 35% had primary education. Of the 40 parents (18 females, 22 males) interviewed, 38 reported frequently self-medicating their children. The data highlighted three major themes: reasons for self-medication, factors influencing self-medication, and most commonly used medication. Parents reported that they self-medicate their children frequently. Most parents kept antipyretics, antibiotics, and cough syrups at home. The majority of the participating parents preferred self-medication due to their lack of trust on healthcare professionals. Factors for self-medication practices included a preference of home-based care, cultural influence, financial and time constraints, the parents' own understanding of the disease and its symptoms, and a lack of basic education.

**Conclusion:**

Pakistani parents in the Abbottabad region frequently resort to self-medication for their children, due to range of factors. The study emphasizes the need of building trust in healthcare experts, raising knowledge and educating people about the dangers of self-medication, and addressing cultural and socioeconomic variables that influence parental decision-making.

## Introduction

Children make up a significant portion of the population in both developed and developing countries (1). The use of medications in children remains a prominent and pressing concern all around the globe (2). Children under the age of five are more susceptible to irrational medications use and its short- and long-term health consequences, making medications use in children a significant public health concern (3). When children become ill, the first reaction of most parents is to self-medicate them (4). The majority of parents in both developed and developing nations prefer to treat their child's common symptoms, such as fever, congestion, and diarrhea, without having to consult a professional doctor or physician (4, 5). Studies have indicated that incorrect self-medication delays medical guidance, worsens health, masks serious diseases, cause medications interactions, bacterial antibiotic resistance, bad medications responses, monetary attrition, and increases the chance of dependency and misuse (6–8). Parents are usually conscious about their children. Such fear and stigma could result in self-medication (9).

According to the World Health Organization (WHO), self-medication occurs when an individual consumes medications based on a self-diagnosis of a disease, without consulting a healthcare professional or conducting clinical tests to validate their assumptions. Economic, political, and cultural factors are responsible for the global prevalence and growth of self-medication. This includes the extensive availability of medications, misleading advertising, the level of education of parents, socioeconomic factors, and healthcare accessibility (10, 11). Self-medication, or the practice of using medications on one's own, is common in both developing and developed nations (12) and has increased in recent years. There has been an upsurge in the practice of self-medication both internationally and locally. A greater incidence of antibiotic self-medication (ASM) among children was reported in the Middle-East (34%), Africa (22%), Asia (20%) and South America (17%), whereas the lowest frequency was identified in Europe (8%) (13).

Based on the latest data, more than 30,015,900 children under the age of 5 are living in the country (14). An estimated 399,429 children in Pakistan died before their fifth birthday in 2021 (15). According to studies conducted in Pakistan, around one-half of parents of children aged 1–5 years old and one-third of parents of children aged 5–12 years old self-medicate their children (16). Study shows that self-medication with antibiotics is most common and frequent in Pakistan (17). In Pakistan, pharmacies may only sell antibiotics with a prescription from a licensed physician (18). However, prior research conducted revealed that 96.9% of pharmacies in Punjab, Pakistan, provided antibiotics without prescriptions (18).

It is essential to understand the factors, reasons, and commonly used medications influencing self-medication, as well as explore parental behavior. Existing literature on this topic is scarce in Pakistan, particularly in the Khyber Pakhtunkhwa (KP) region. The qualitative aspects of self-medication in this area remain unexplored, which would provide valuable insights into this issue. Additionally, long-term use exacerbates the risk of adverse events in children, which parents often do not understand. This research would be helpful for public health experts to develop effective policies to curb self-medication among this age group. Therefore, the current study was aimed to explore the Pakistani parents' perceptions and behaviour towards self-medicating their children, and the reasons of parental self-medications in Abbottabad, Pakistan.

## Methods

### Study design

This exploratory study used observational qualitative methods to investigate parental attitudes and practices regarding children's self-medication. To select the participants, a non-probability method of convenient sampling was utilized. Individual in-depth interviews with participants, recruited from different parts of the Havelian, were conducted to collect data. In addition, individual in-depth interviews, were beneficial for gaining a deeper understanding of the research objectives and gave respondents more time to communicate.

The research was conducted in different parts of the Havelian, a tehsil of the city of Abbottabad, Khyber Pakhtunkhwa Province, Pakistan. It is the second-largest municipality in Abbottabad District, located along the Karakoram Highway at an elevation of 3,081 feet with a total population of 40,481. In the city, there is one public hospital along with several private hospitals and clinics (19).

### Study population

The study population were the parents of children under the age of five who resided in the selected geographical region. Participants were either biological mothers or fathers of children. A total of 60 parents were approached, out of which 40 agreed to participate in the study.

Data collection and participant recruitment were conducted through site visits to pharmacies, clinics, and hospitals, where researchers identified and approached potential participants. Parents visit individual pharmacies, clinics, and hospital pharmacies to obtain medicine. Short message service messages explaining the objectives and methodologies of the study were sent to the mobile phones of 60 study participants, out of which only 40 replied and agreed to participate and information saturation was obtained till the last participants. The response rate was 67%. Those participants who were agreed were visited door-to-door and informed about the study on the spot. They were informed and assured of the confidentiality and anonymity of their interviews.

### Inclusion criteria and exclusion criteria

Parents who were residents of the Havelian, agreed to sign the informed consent, and had children under the age of 5 years were considered to participate in the study.

Parents who had communication issues, or didn't have children under 5 years of age, were excluded from the study.

### Interview guide about self-medication

Interviews were conducted by trained researchers, who were trained by the principal investigator prior to the interviews. The principal investigators, with their vast experience in qualitative studies, have trained the researcher. In-depth interviews ranging from 45 min to 1 h and 30 min were conducted at the place and time of respondents’ choice for their convenience and result's accuracy. During the interview session, answers of respondents were audio-recorded, properly written down and key points were captured. Before conducting the interview, parents were given insight into the basic understanding of the term “self-medication” and what type of questions were included in the study/interview. The interviews were carried out in the Urdu and local language Hindko. The responses were transcribed into English for thematic analysis. The first few questions of the interview focused on socioeconomic variables such as age, sex, profession, educational level, income, number of children and their ages. The next question we asked was, “Have you given your child any kind of medications in the past three to six months?” If they responded in the affirmative, we went on to ask more detailed questions about self-medication, including the circumstances under which parents preferred to self-medicate, the medicines that were used, the source of information, the duration of treatment, the results of self-medication, and other general perceptions that participants had regarding self-medication. Depending on the answers of parents we asked more detailed and precise questions to obtain all the relevant information. Interview guide has been given in Supplementary File 1. This guide was pretested before starting research and data was not included in the thematic analysis.

### Data analysis

The interviews' audio files and documentation were organized and compiled using Microsoft Word, and transcripts were subsequently generated. To mitigate the potential influence of researcher bias, the interview transcripts were disseminated to respondents for their review and validation. Thematic analysis was performed with the aid of NVivo 11.0, a software designed for the analysis of qualitative data. Data has been presented according to COREQ checklist (Supplementary File 2). Themes and sub-themes were identified and generated from the transcripts using NVivo 11.0. A team of three authors independently decided on important themes and matching subthemes after reviewing the transcripts and audio files numerous times. Later, researchers used an interactive discussion approach to develop the thematic framework and finalized the mentioned themes.

### Ethical considerations

This study was based on the ethical guidelines of the Declaration of Helsinki and received approval from the Research and Ethics Committee of The University of Haripur, Khyber Pakhtunkhwa, Pakistan (UOH/ASR/23/1370). Before commencing the interview, the aim and approach of the study were shared with respondents. Parents were also made aware of their autonomy to withdraw from the research at any moment. Written and informed consent for involvement and permission for audio-recording were acquired.

## Results

**Figure 1:**
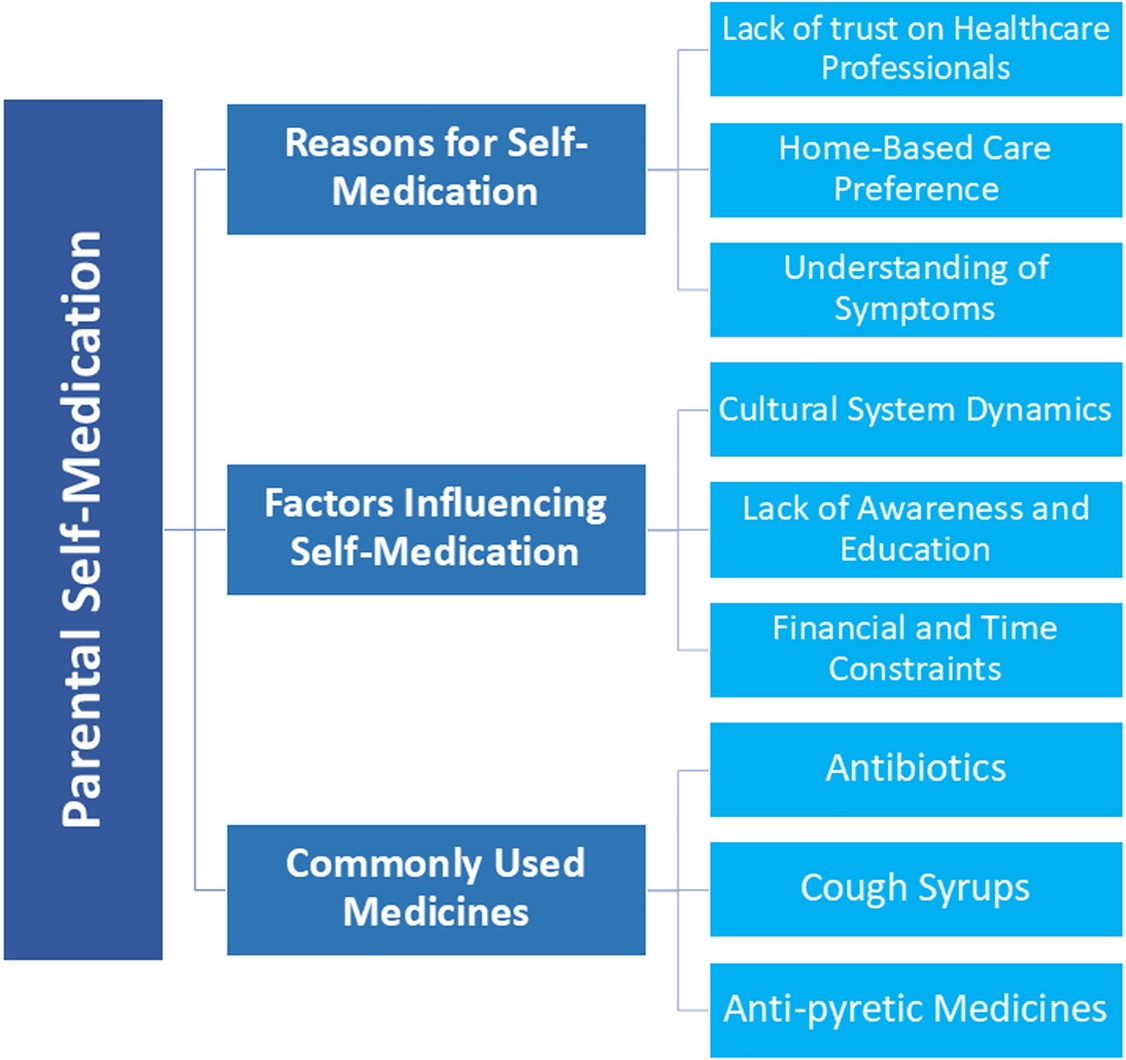
Main theme and their sub-themes.

Fourty parents were interviewed (Females = 18, Males = 22), out of which 38 parents frequently self-medicated their children while 2 of them only opted for self-medication when felt necessary (Table 1). The majority of parents had antipyretic medications, common antibiotics and cough syrups available at home most of the time. The majority of parents ranged in age from 25 to 35 years old (45%), and most of them only had primary level of education (35%). Out of total 18 females, 13 of them were housewives (45%) with only one being a school teacher in profession as shown in Table 2. The study's findings unveiled three main themes: Reasons for self-medication, factors influencing self-medication, and commonly used medications. The main themes were classified into various corresponding sub-themes, as illustrated in Figure 1.

**Table 1:** General information of the study respondents.

Codes	Gender	Age	Profession	Monthly income	Education level	Number of children
R1F	Female	40	Housewife	50,000–100,000	Secondary	4
R2M	Male	44	Govt. Teacher	50,000–100,000	Tertiary	5
R3F	Female	35	Housewife	Less than 50,000	Primary	2
R4M	Male	52	Businessman	More than 100,000	Tertiary	3
R5M	Male	39	Property Dealing	More than 100,000	Primary	3
R6M	Male	42	Fruit Vendor	Less than 50,000	Secondary	4
R7M	Male	55	Local Businessman	More than 100,000	Tertiary	2
R8F	Female	26	Housewife	50,000–100,000	Secondary	2
R9M	Male	38	Clerk + Side Businesses	50,000–100,000	Tertiary	5
R10M	Male	31	Businesses (Property, Farms)	More than 100,000	Tertiary	4
R11F	Female	28	Housewife	More than 100,000	Primary	2
R12M	Male	43	Property Dealing	More than 100,000	Secondary	3
R13M	Male	27	Gym Owner	50,000–100,000	Primary	2
R14F	Female	30	Housewife	50,000–100,000	Primary	2
R15F	Female	28	School Teacher	50,000–100,000	Tertiary	3
R16F	Female	35	Housewife + Sewing	50,000–100,000	Primary	2
R17F	Female	50	Housewife	Less than 50,000	Primary	5
R18F	Female	39	Housewife	Less than 50,000	Secondary	2
R19M	Male	42	Electrician	Less than 50,000	Secondary	4
R20M	Male	37	Shopkeeper	Less than 50,000	Primary	3
R21M	Male	32	Daily wage worker	Less than 50,000	Primary	1
R22F	Female	29	Housewife + Tutor	More than 100,000	Tertiary	3
R23M	Male	41	Private Businesses	More than 100,000	Tertiary	2
R24F	Female	33	Govt. Employee	50,000–100,000	Secondary	2
R25M	Male	40	Businessman	More than 100,000	Secondary	4
R26F	Female	36	Housewife	Less than 50,000	Tertiary	3
R27M	Male	23	Street Vendor	Less than 50,000	Primary	2
R28F	Female	25	Lady Health Visitor	50,000–100,000	Tertiary	1
R29M	Male	55	Businessman	More than 100,000	Tertiary	3
R30F	Female	34	Housewife	50,000–100,000	Primary	2
R31M	Male	32	Plumber	Less than 50,000	Secondary	3
R32F	Female	40	Businessman	More than 100,000	Tertiary	4
R33M	Male	40	TMA Employee	50,000–100,000	Primary	2
R34F	Female	29	Housewife	Less than 50,000	Secondary	3
R35M	Male	21	Shopkeeper	Less than 50,000	Tertiary	1
R36F	Female	26	Housewife	More than 100,000	Tertiary	2
R37M	Male	44	Businessman	More than 100,000	Secondary	5
R38F	Female	36	Home Caretaker	Less than 50,000	Primary	2
R39M	Male	28	Mason	Less than 50,000	Secondary	4
R40M	Male	51	Farmer	Less than 50,000	Primary	3

**Table 2:** Demographic information of the study respondents.

Variables	Frequency	Percentage
Gender		
Male	22	55%
Female	18	45%
Age		
25–35	18	45%
36–45	17	42.5%
>45	5	12.5%
Profession		
Housewife	13	32.5%
Businessman	10	25%
Daily wage worker	8	20%
Govt/Pvt employee	9	22.5%
Monthly income		
Less than 50,000	15	37.5%
50,000–100,000	12	30%
More than 50,000	13	32.5%
Education level		
Primary	14	35%
Secondary	12	30%
Tertiary	14	35%

### Reasons for self-medication

#### Lack of trust in healthcare professionals

95% of the parents (*n* = 38) favored self-medication because they felt that doctors and other healthcare professionals are not trustworthy enough. According to some parents (*n* = 10), doctors fail to communicate effectively or show empathy towards patients by being straightforward and assertive.

“I don't solely rely on doctors and medicines because medicines nowadays are causing so many side effects and the medicines bring 2 other new problems along with the cure”, according to one parent (R1F).

Parents also stated that they're not being educated about the adverse effects of self-medication of children by doctors. According to a parent,

“Doctors are not usually interested in educating us on this issue. They just ask us about the basics of illness/disease of the children but don't emphasize on rights and wrongs of self-medication” (R21F)

In addition to their lack of confidence in healthcare professionals, parents in this study reported unpleasant experiences with government hospital staff. In particular, many parents complained about the unfriendly and impolite staff, which contributed to their overall dissatisfaction with the healthcare system.

“Staff at government hospitals are not friendly and are rude most of the time” (R2M)

#### Home-Based care preference

20 out of 40 parents stated that treating their children at home with remedies and avoiding medical care and physicians was their primary concern. If the condition worsened, however, they would consult a specialist. In addition, the parents revealed that in their communities, treatment at home with remedies and medicines was preferable and that they would consult with one another to gain a better understanding. When a family member was ill, one parent stated that the family had always relied on household remedies rather than pharmaceuticals or medical professionals. However, they would only visit a doctor when necessary; otherwise, they relied on self-medication.

“I come from a family that has always relied on home remedies rather than pharmaceuticals or medical professionals when someone in the family was sick. In the past, we would see a doctor only if the situation warranted it; otherwise, we relied on self-medication.” (R35M)

“My first priority usually is to treat my kids at home with remedies and avoid medical care and doctors” (R1F)

“Here in our communities we prefer to treat at home by remedies and medicines and discuss with each other for better understanding” (R7M)

These choices reflect the social and cultural norms of parents, which may influence the parents' health-seeking behavior and health outcomes.

#### Understanding of symptoms

Significant numbers of parents (*n* = 14) self-medicated their children based on their own perception and understanding of symptoms. Parents self-medicated because they believed they comprehended their child's symptoms of illness. One parent stated

“At first I used to go to the proper doctor and ensure treatment but now I have a grip on the symptoms of my child's illnesses so I am now confident in medicating my child” (R4M). Another parent stated that “I usually see if the symptoms are recognizable then I prefer self-medication otherwise I consult a professional” (R10M).

Past experiences with other children also contributed to parent's perception and comprehension of the child illness and symptoms. A male parent, when ask about whether past experience with their other children's illness helped in understanding symptoms, responded,

“Certainly it is impacted by previous experiences. My older children have been unwell so frequently in the past that I now have the knowledge and experience to treat and self-medicate my younger children without seeing a doctor” (R11M).

### Factors influencing self-medication

#### Cultural system dynamics

Traditional cures and self-care practices are profoundly rooted, and parents (*n* = 9) frequently relied on them to treat minor afflictions.

“Cultural notion influences my decision to self-medicate my kids and my health care views” (R3F)

For the majority of parents, the opinion of family and community members held great importance, which also played a role in self-medication.

“Young parents like me self-medicate because of our combined family system. Other family members with experience influence our decision of medicating the child for certain disease” (R8F)

“I also ask my family members and friends for advice” R6M

Female parents, due to their religious and cultural beliefs, didn't like to attend doctors on their own unless accompanied by their husbands or another family member.

“My husband isn't usually around, he works in Islamabad. So there isn't anyone I can rely on and visit the doc. I can't visit the hospitals and doctors by myself. I don't own a vehicle or car, my kids are young and I am all by myself” (R1F)

“I don't have a worker/maid at home and my husband is not at home most of the daytime, so these are the basic reasons for self-medication” (R8F)

#### Lack of awareness and education

Out of total 40 parents, 35% had a primary level of education while 30% had secondary and the remaining had at least a bachelor's degree as shown in Table 2. 10 parents mentioned that they are confident in self-medicating as they have appropriate knowledge about the dosage and duration of medicines.

“Now I am much more experienced and have appropriate knowledge cuz I have now 5 kids”. (R2M)

“I have some knowledge about medicine and healthcare from my own experiences and from talking to family members and friends, so I feel capable of managing my child's health on my own”. (R36M)

“My husband also helps with such actions, he has some knowledge of medicines and time duration of medicines for children” (R8F)

30 parents didn't have any negative experiences regarding the self-medication of their children and believed this action to be a beneficial and better option than doctors and hospitals.

“It (self-medication) is a good thing if you are confident in your decision and have a knowledge of disease and its knowhow” (R4M)

“I had quite some unpleasant experiences with doctor visits. I’ve been self-medicating since then and had no bad experience so far” (R17F)

“Parents are not aware of the dangers of self-medication and those who are aware, do not have enough resources to consult professionals for their child's illness.” (R16F)

#### Financial and time constraints

The cost of healthcare was identified as a major impediment to obtaining professional medical assistance, with many parents claiming they don't have the means to pay for doctor visits or medicines. 67% of the parents either had monthly income ranging from 50,000 to 100,000 PKR or less than 50,000 PKR (Table 2) making it difficult for them to properly manage the cost and bills of healthcare facilities. 16 out of 40 parents mentioned affordability as their main reason for self-medication.

“People who can afford to see doctors often consult them but a lot of people who can't afford the fee, like me, depend on home remedies and self-medication” (R18F)

“I self-medicate my child because it is often the only option I have as a low-income parent” (R6M)

“Medicines and doctor appointments these days are so expensive that it takes up a big chunk of money for a single visit to a clinic or hospital” (R8F)

One parent stated that they had no choice but to take care of their child's minor illnesses themselves, even though they may not have the necessary knowledge or expertise to do so safely and effectively.

“If I didn't take care of my child's minor illnesses myself, I would have to let them go untreated or pay money I don't have to a doctor or nurse” (R3F)

Parents who had previously experienced positive outcomes with self-medication were more likely to continue the practice, indicating that it is a widely accepted and trusted approach to healthcare in this population.

“Self-medication is prevalent, as it is handy, inexpensive, and quick to obtain” (R12M)

Numerous parents (*n* = 16) stated time and convenience as reasons for self-medication. Most of them were on full-time job, like shopkeepers (R20M) and vendors (R6M), who couldn't afford free time due to the nature of their work.

“People like me that are busy most of the daytime will prefer the easiest option available and that seems to be self-medication” (R9M)

“Mostly in day time and in the mornings doctors are highly rushed and busy, you’ll have to get an appointment in the morning but your turn will be at noon or evening. So parents like us prefer to treat at home rather than these busy places” (R14F)

The availability and accessibility of hospitals were the primary determinants of convenience for parents. The distance between their home and the closest healthcare facility made medical stores and self-medication more accessible and handy.

“Yes after he (child) got sick we took him to a nearby store because the hospital was at quite some distance, and the store attendant prescribed some basic fever syrups and one injection for allergy” (R3M)

“We mostly prefer medical shop vendors for the sake of convenience. We explain the problem, and he provides the necessary medications”. (R11F)

### Commonly used medications

#### Antibiotics

14 out of 40 parents (35%) were found to be administering antibiotics on a frequent basis.

“Yes once my child had fever so I gave him antibiotics but it got worse and severe stomach ache started so I panicked and rushed to doctor” (R1F).

“My toddler had diarrhea and a high fever last month, so I gave him antibiotics and a fever-reducing syrup” (R10M).

#### Cough syrups

40% of parents had cough syrups available at home most of the time.

“My younger son had a fever and a cough a few months ago. I gave him some medicine that I had at home, like paracetamol and cough syrup” (R26M).

“Medications for gastrointestinal disorders, fever, cough, allergy, stomach discomfort, etc. are often accessible to us parents, typically moms, at home” (R11F)

#### Anti-pyretic medications

A significant number of parents utilize over-the-counter medications to manage their child's fever and related symptoms. It was found that 55% of parents (22 out of 40) used antipyretic medications and syrups to treat their child's fever and body aches. The most commonly used medications were paracetamol and ibuprofen.

“I did give my child Tylenol to treat her fever” (R20M)

“Yes I do consult someone with experience of children. But usually for fever I’ll give ibuprofen or any antipyretic syrup for the time being” (R1F).

“My youngest kid had a fever and a cough. To relieve his discomfort, I gave him some paracetamol and herbal tea” (R39F)

## Discussion

The current study revealed self-medication to be a common practice among parents of young children with major reasons being; financial and affordability issues, time constraints, lack of trust in healthcare professionals, cultural beliefs and dynamics, reliance on remedies and home-based care, and lack of awareness.

A lack of trust in healthcare professionals was one of the most prevalent causes of self-medication with 90% of the parents mentioning it as the main reason for self-medication of their child. Some parents reported that physicians were ineffective communicators and lacked empathy for patients because they were direct and assertive. The parents in the study believed that physicians and other healthcare professionals lacked sufficient trustworthiness, and their interactions with healthcare professionals were unsatisfying. This is similar to the results of a study conducted by (20), in which a lack of trust in healthcare system was mentioned as one of the reasons for self-medication. A significant proportion of parents administer self-medication to their children based on their own assessment and interpretation of their children's symptoms. This practice is usually affected by the parents' prior experiences dealing with the ailments of their children. This is in accordance with the previous studies of (1, 16) which revealed that parents practiced self-medication because they believed that if they visited the doctor, the prescription would be the same as prescribed previously by a doctor for the same ailment.

Our study also highlighted the cost of healthcare as a significant barrier to obtaining professional medical treatment, in addition to the waiting time at the hospitals and clinics, as many parents reported that they lacked the financial resources to pay for doctor visits or to wait in line for hours in hospitals and clinics. This was one of the major findings of the study. This is in agreement with the previous studies (4): which state waiting times in clinics and hospitals and expensive consultation fees as the main reason for parental self-medication. Some parents reasoned their use of self-medication by claiming that their excessively hectic work schedules prevented them from seeing a physician and so they did not have the time to do so; this is in agreement with the study of (16) which explains that parents self-medicated their child because they could not afford enough time to pay a visit to doctor/physician due to their work-load. Many parents who had positive experiences with self-medication continued the practice, some acknowledged the risks and perils associated with self-medication, particularly when done without the necessary knowledge and expertise. It was observed that parents were unaware of the potential hazards of self-medication. A study in India also reported similar results in which significant proportion of respondents were not aware of possible adverse effects of self-medication (21). Cultural and conventional beliefs exerted a large amount of impact on the health-seeking behavior of the parents. Traditional medicines and home-based remedy care were deeply embedded in the lives of many parents, and they regularly turned to these approaches to treat their children's minor diseases (22). The current study also revealed that the perspectives and opinions of members of the family and the community were highly appreciated by the parents and played a substantial part in the practice of self-medication.

The study revealed that parents in the community favored treating their children with remedies and herbal medicines at home. This predilection for home remedies and traditional medicines may have led parents to medicate their offspring on their own which is in agreement with a previous study (23). However, the study findings also indicated that parents relied on self-medication only when the condition was not severe and that they would consult a specialist if the condition worsened. This indicates that some parents were aware of the limitations of self-medication and willing to seek medical assistance as necessary. This finding of the study is similar to the previous study by (1) which indicates that parents self-medicated if the disease was simple and would only consult a health professional if the child does not recover from the ailment. The most frequent medicines used by parents for self-medication were Anti-pyretic medications 55%, cough syrups 40% and antibiotics 35%. As parents were unaware of the inadequate antibiotic use or its proper duration, it may lead to the development of antimicrobial resistance in their children (24).

### Limitations

The small sample size of parents was one of the limitations of the study. This sample size may not be a representative of the larger population and is limited to one region of the province, which may restrict its generalizability to the entire population. Future research targeting a broader population from diverse regions would be beneficial, providing a more comprehensive understanding of the issue. Another limitation of the study was that people of the KPK region are reluctant to participate in research studies due to cultural stigmas. Females are often not allowed to speak in public or engage in any kind of public activities on free will, and as a result, the study had a disproportionate sample of males, with only 45% of the participants being female. This gender disparity may have biased the results of the study. There is a need for increased awareness among people so that more female may participate in research and future studies can be more authentic and veritable. Another limitation of our study is self-reported behaviors, which may not always be accurate as with most qualitative studies.

## Conclusion

The study revealed that parents lacked knowledge and that their self-medication practices were inappropriate. The prevalent use of parental self-medication in Havelian region of northern Pakistan should be regarded as a dire concern. Since the majority of parents are unaware of the adverse health effects of self-medication, this can contribute to medication abuse and subsequent problems such as antibiotic resistance. The leading causes of parental self-medication were financial constraints, lengthy waiting times in hospitals, and lack of knowledge regarding disease or its symptoms. It was also noted that parents, for their convenience, consulted the local pharmacist for the recommendation of medicines. There is a crucial need for suitable interventions to address this issue. Enhancing communication and empathy of healthcare professionals towards parents, along with improved healthcare accessibility, parental education on responsible medication use, addressing social norms, and promoting cultural sensitivity, can foster safer healthcare practices and improved health outcomes for children in the Havelian region, KP Pakistan. Interventions should also include awareness of parents regarding self-medication consequences for their children through involvement of academia along with health department for better outcomes.

## Data Availability

The original contributions presented in the study are included in the article/Supplementary Material, further inquiries can be directed to the corresponding author.
