# Supplementary material for: A qualitative exploration of parental perspectives and behaviors on self-medication for children under five in Abbottabad, Pakistan
Source: Front Pediatr. 2025 Apr 16;13:1445219. doi: 10.3389/fped.2025.1445219 (PMC12040883; doi:10.3389/fped.2025.1445219)
Supplement: Supplementary file 2 [file Datasheet2.pdf]

Unstructured Open-ended Interview

Hanzila Azhar

Basic Info

1. **Age**	
2. **Gender**	
3. **Educational status**	
4. **Monthly income**	
5. **Number and age of children**	
6. **Profession**	
7. **Medical Insurance**	

Discussion with Parents

- In the last 3-6 months did you child suffer from any kind of illness or disease? If YES than what methods did you use for treatment?
- In the last 3– 6 months did you give any type of drug to your child?
- Do you feel comfortable making decisions about your child's healthcare, or do you prefer to consult a healthcare professional?
- What factors do you consider when deciding whether or not to self-medicate your child?
- Have you ever had any negative experiences or concerns related to self-medication of your child?
- How do you usually obtain information about appropriate medications and dosing for your child?
- Do you think that self-medication is a common practice among parents of young children? If so, why do you think that is?
- How do you think healthcare professionals, such as pharmacists or pediatricians, can help to educate parents about appropriate medication use for children?
- Do you think other parents in your community self-medicate their children as well? Why or why not?
- Can you share any personal experiences or stories about the consequences, positive or negative, of self-medicating your child?
- In your opinion, when is it appropriate for a parent to self-medicate their child? When is it not appropriate?
- How do you think your child's healthcare might be different if you did not self-medicate?
- Do you think your self-medication practices are influenced by your own healthcare experiences or beliefs? Can you tell me more about how your own healthcare experiences may have shaped your decision to self-medicate your child?
- What role do you think cultural or societal factors play in your decision to self-medicate your child? Do you think your self-medication practices are influenced by your cultural or societal background?

Generalized Questions

- Do you know about Self Medication?
- Have you ever taken Self Medication?
- Have you taken self-medication in last three months?
- Did your child suffer from any disease in the last 3 months?
- Have you self-medicated your child in the last three months?
- What was your reason for self-medication (for child)?
- What do you consider while selecting the drug for self-medication?
- Where do you obtain your drugs for self-medication?
- How much do you understand about drugs or antibiotics?
- Have you ever self-medicated yourself with anti-biotic?
- How long you have been practicing self-medication on your child?
- For which common disease do you self-medicate your child?
- How do you know the dosage of drugs/antibiotics that is suitable for your child?
- Did you ever change the dosage of drugs/antibiotics during the course of self-medication?
- Which drug do you use most often for self-medication?
